# Disability-inclusive reproductive healthcare: A reproductive health education intervention for caregivers of girls with cerebral palsy in India

**DOI:** 10.1016/j.puhip.2026.100855

**Published:** 2026-09-16

**Authors:** Chithra Babu, Anusree Prabhakaran, Hitesh Shah, Arathi P. Rao

**Affiliations:** a Department of Global Public Health Policy and Governance, Prasanna School of Public Health, Manipal Academy of Higher Education, Manipal, India; b Department of Paediatrics Orthopaedics, Kasturba Medical College, Manipal Academy of Higher Education, Manipal, India

**Keywords:** Caregiver intervention, Cerebral palsy, Disability inclusion, Menstrual hygiene, Reproductive health education, Sustainable development goal 3

## Abstract

**Objective:**

Girls with Cerebral Palsy (GCP) face unique difficulties during adolescence, particularly related to menstruation and reproductive health, which are compounded by caregivers’ limited knowledge and preparedness. Educating caregivers may improve the management of menstrual and reproductive health concerns in GCP. Hence, this study was conducted to assess the effect of a Reproductive Health Education (RHE) intervention on knowledge and reproductive health-related caregiving intentions among caregivers of GCP in India.

**Study design:**

A single-arm pre-test-post-test pilot intervention study.

**Methods:**

The study was conducted among caregivers of GCP attending a weekly CP clinic at a tertiary care hospital in South India. Participants were recruited consecutively within a predefined time frame. The intervention consisted of a one-on-one education session, guided by a validated Information, Education and Communication (IEC) material. Knowledge and reproductive health-related caregiving intention were assessed before and 3 months after the intervention using a structured questionnaire. Paired t-tests were used to compare outcomes before and after the intervention.

**Results:**

A total of 25 caregivers were included. A significant improvement in both knowledge and caregiving intention was observed following the intervention. Mean knowledge score increased from 41.2 ± 4.66 to 62.3 ± 1.77 (p < 0.001, 95% CI of mean difference = 19.5-22.7), and the mean caregiving intention score rose from 41.2 ± 4.02 to 57.6 ± 1.78 (p < 0.001, 95%CI of mean difference = 15.1 -17.7). The corresponding paired Cohen's d value was larger (Cohen's d = 5.43 for knowledge; 5.22 for caregiving intention).

**Conclusion:**

The study provides preliminary evidence on the effect of a brief tailored RHE intervention on knowledge and reproductive health-related caregiving intention among caregivers of GCP. Causal inference is limited by the single-arm design of the study.

**What this study adds?**

• Provide context-specific evidence from a lower-and middle-income country, addressing a neglected area of disability-inclusive reproductive health care.
• Shows pre-post increase in knowledge and caregiving intention after a brief reproductive health education among caregivers of girls with cerebral palsy.

**Implications for policy and practice**

• If confirmed in a controlled study with larger sample, a brief reproductive health education session, integrated into routine cerebral palsy consultations may be a practical way to address caregivers' information needs in low resource settings.

## 1 Introduction

The United Nations estimates that 240 million children worldwide live with some form of disability. [1] Cerebral Palsy (CP) is one of the most common disabilities affecting infants and children globally, with an estimated prevalence of 2 to 2.5 per 1000 live births. [2,3] In Low-and Middle-Income Countries like India, the burden of CP is more pronounced, with a pooled prevalence of 2.95 per one thousand children. [4]

CP is a lifelong neurodevelopmental disability resulting from damage to the developing brain that primarily affects a child's movement and posture. [5] Children with CP often experience varied difficulties, including motor impairments that affect walking and independence. Many also have other conditions such as speech difficulties, feeding problems, sensory impairments, and intellectual or behavioural challenges, which may affect their daily functioning and self-care. [2,[6], [7], [8], [9]]

As they reach adolescence, additional challenges emerge, especially in girls with CP (GCP). Menstruation and pubertal changes may pose significant difficulties to the GCP due to their physical limitations, difficulties in menstrual hygiene management and increased vulnerability to abuse. [[10], [11], [12]] Communication barriers may further complicate the recognition and management of menstrual pain, discomfort or other reproductive health concerns. [11] Findings indicate that these girls often need assistance from their caregivers during menstruation. [11] Caregivers, therefore, play a central role in supporting the GCP's menstrual and reproductive health needs. Studies have reported that caregivers lack training, support and knowledge to manage menstruation-related issues of GCP. [[13], [14], [15]] Strengthening caregivers' knowledge and skills is essential to support the reproductive health and well-being of GCP. Existing studies from India have explored the experiences and difficulties faced by caregivers in managing menstruation and reproductive health issues of GCP and other developmental disabilities. [[14], [15], [16], [17]]

Rao et al. (2019, 2021) explored menstruation-related issues from the perspectives of GCPs and caregivers in tertiary hospital settings in India. Their findings described significant caregiver anxiety, discomfort in discussing reproductive health, and the absence of formal support systems. [12,15] Although these studies provide existing evidence on the challenges faced by caregivers of GCP, there is limited research on practical, implementable interventions that can be integrated into routine CP care, particularly in Lower- and Middle-Income Countries (LMICs). Existing intervention studies primarily focused on caregivers of girls with intellectual disabilities and were not specific to cerebral palsy. In Iran, Goli et al. found that group training was effective for sexual healthcare-related awareness, attitude and self-efficacy of mothers of intellectually disabled girls. [18] Similarly, in Turkey, Kacan and Birol evaluated a menstrual self-care program among girls and caregivers with intellectual disability and reported positive effects. [19] Although these studies provide evidence on the importance of caregiver education, the focus on caregivers of girls with intellectual disability limits the generalizability of the findings to caregivers of GCP, who may have distinct caregiving needs related to motor limitations, communication difficulties, and dependence during menstrual care.

Draz and Elsharkawy (2020) evaluated a supportive educational intervention delivered to caregivers of GCP at the outpatient clinic in Egypt and reported improvement in caregiving practices. [20] However, the intervention and outcome assessment primarily focused on selected aspects of routine and perineal care, with less emphasis on the caregivers’ knowledge required to support appropriate reproductive health-related caregiving. Thus, despite the evidence that reproductive education improves certain aspects of caregiving, there remains limited evidence on a caregiver-targeted educational intervention for GCP that specifically strengthens caregivers' reproductive health knowledge and their intention to provide appropriate menstrual care. Addressing this gap is critical to ensure the caregivers are equipped with the necessary knowledge to handle the reproductive health-related concerns of GCP. This study was therefore conducted to address this gap by assessing the effect of a reproductive health education intervention, integrated into the routine CP care, on the knowledge and caregiving intentions of caregivers of girls with cerebral palsy. This study is novel in that it specifically focuses on caregivers of girls with cerebral palsy and addresses their reproductive health-related knowledge and caregiving intentions through a reproductive education integrated into the routine cerebral palsy care of the child.

## 2 Methods

### 2.1 Study design

A single-arm pretest–posttest pilot study was conducted to assess the effect of a reproductive health education intervention on caregivers of GCP.

### 2.2 Study setting and study period

The study was conducted within the existing clinical workflows of the CP clinic, which operates under the Department of Paediatric Orthopaedics at a tertiary care referral hospital in South India. The clinic operates every Thursday and caters to children and adolescents with CP. The study was conducted between January and June 2025.

### 2.3 Sampling and participants

The sample size was determined by the number of eligible participants attending the weekly clinic during the study period. A consecutive sampling method was utilised to recruit the participants. Twenty-five participants, irrespective of their GCP's menarche status, were included in the study. Caregivers who were directly involved in the daily care of the GCP and attended the CP clinic during the study period were considered eligible and invited to participate. Recruitment was conducted during their outpatient (OP) visits to the CP clinic. Participants were provided a copy of the Participant Information Sheet (PIS) and were informed about the study prior to enrolment. Participation was voluntary, and written informed consent was obtained from each participant prior to their participation.

### 2.4 Intervention

The Reproductive Health Education intervention topics generally covered menstruation, menstrual hygiene, menstrual pain management, body changes associated with puberty, reproductive safety, and the reproductive rights of GCP. The intervention was delivered by researcher through a brief face-to-face educational session. A validated, previously published Information, Education, and Communication (IEC) booklet containing visual illustrations for better understanding along with simple text was used to guide the sessions. The booklet was originally developed in English and later translated into the local languages, Kannada and Malayalam, to facilitate better understanding among participants. The intervention was delivered by the researcher to all study participants in a one-on-one format, enabling personalised interactions and clarifying concerns. It was delivered in a designated room within the CP clinic at the tertiary care referral hospital by the primary researcher, who is a trained public health professional. Each participant received a single educational session, lasting approximately 20-25 min. Following the educational session, each participant was given the opportunity to ask questions and clarify their concerns about the management of their child's reproductive health and related issues. All queries were addressed by the primary investigator. In addition, each participant was provided with a printed copy of the IEC booklet for future reference. To minimise researcher-related bias and variability in intervention delivery, the intervention was delivered strictly according to protocol: the researcher delivered the intervention consistently and uniformly to each participant, following the same predefined content, sequence, duration and instructions and only participant-initiated queries or doubts were addressed, and no additional information was selectively provided.

### 2.5 Data collection instrument

A structured questionnaire, developed in accordance with the IEC booklet, was used to assess the knowledge and reproductive health-related caregiving intention of caregivers of GCP. It consisted of questions about the participants' socio-demographic characteristics, the demographic and clinical characteristics of the GCP, knowledge, and caregiving intention related to menstruation, menstrual hygiene management, reproductive safety, and the reproductive rights of GCP. There were 13 items for knowledge and 12 items for caregiving intention, each rated on a 5-point Likert scale. Total scores were calculated and analysed to assess changes in knowledge and caregiving intention before and after the intervention. The tool was content-validated by subject-matter experts in public health and disability care to ensure its relevance, clarity, and appropriateness for the target population. Internal consistency of the tool was assessed using Cronbach's alpha. For the knowledge domain, Cronbach's alpha was 0.602 at pre-test and for the caregiving intention domain, Cronbach's alpha was 0.612 at pre-test. Post-test reliability could not be estimated because several items had zero variance (ceiling effect).

### 2.6 Data collection and follow-up

Baseline data were collected by administering the questionnaire just before delivering the RHE intervention (pre-test). Immediately after the pre-test data collection, the intervention was delivered to the participants. Participants were reminded to refer to the booklet during the follow-up period and were advised to contact the primary investigator if they had any doubts or queries. Post-intervention, data were collected using the same structured questionnaire via a telephonic call at a three-month follow-up. To reduce social desirability bias, participants were told that the information would be kept confidential and that there were no right or wrong answers. In addition, to reduce measurement biases, the outcome assessment was based on a structured questionnaire with predefined scoring criteria rather than on subjective researcher judgment. Also, the researcher did not probe or influence the participants’ responses.

### 2.7 Outcome measures

Knowledge: Caregivers’ understanding of menstruation and menstruation management of GCP.

Caregiving intention: Caregivers' self-reported intentions to adopt practices that support menstrual hygiene and ensure the GCP's reproductive safety and rights.

Operational definition of reproductive health: In this study, reproductive health refers to aspects related to menstruation, menstrual hygiene management, management of menstrual problems, and reproductive safety and rights of GCP.

### 2.8 Statistical analysis

Data were entered into Microsoft Excel and analysed using JAMOVI 2.3.28 software. Descriptive statistics were used to summarise sociodemographic and clinical characteristics. Paired t-tests were used to compare pre- and post-intervention scores for both knowledge and caregiving intention. The total scores for the individual items within the knowledge and caregiving intention domains were first summed for each participant to obtain an overall domain score. Subsequently, the mean and standard deviation of these total scores were calculated separately for the pre-test and post-test assessments. For the paired pre–post comparison, effect size (Cohen's d) was calculated based on the mean change between the pre-test and post-test scores and the standard deviation of the paired differences. Statistical significance was set at p < 0.05. An item-wise analysis was also conducted to identify specific areas for improvement.

## 3 Results

### 3.1 Socio-demographic characteristics of the participants

A total of 25 caregivers who met the inclusion criteria and provided consent to participate were included in the study. All 25 participants completed the pre-intervention assessment, received the reproductive health education intervention and completed the follow-up. The mean age of caregivers was 39 years, and the majority were mothers (72%), unemployed (72%), resided in urban areas (68%), and had completed at least secondary education (44%). All participants were the primary caregivers for their children and had provided care for more than 10 years. The socio-demographic characteristics of the participants are summarised in Table 1.

**Table 1:** Sociodemographic characteristics of the participants.

Variable	Category/Value	Frequency	Percentage
Age group (in years)	31-35	6	24%
	36-40	7	28%
	41-45	6	24%
	46-50	4	16%
	51-56	2	8%
Gender	Female	18	72%
	Male	7	28%
Relationship to the child	Mother	18	72%
	Father	7	28%
Education Level	Illiterate/Primary	1	4%
	Middle School	4	16%
	High School	6	24%
	Intermediate/Diploma	6	24%
	Graduate	5	20%
	Postgraduate	3	12%
Occupation	Employed	7	28%
	Not Employed	18	72%
Marital Status	Married	25	100%
Household income/month in rupees	< 15,000	6	24%
	15,000–40,000	13	52%
	40,001–100,000	4	16%
	> 100,000	2	8%
Primary/Secondary caregiver	Primary	25	100%
Duration of Caregiving (in years)	<5	2	8%
	5-10	6	24%
	11-15	14	56%
	>15	3	12%

### 3.2 Demographic and clinical characteristics of the GCP

Among the GCP, 60% were aged 12 years or older, and 40% were in Grade 3 of the Gross Motor Function Classification System (GMFCS) [21]. IQ scores ranged from 50 to 90, with 40% of children having mild intellectual disability (IQ 71–80). All children were receiving ongoing care for CP, and no co-existing medical conditions were reported. Nearly all caregivers (96%) reported some improvement in their child's motor skills following therapy. The demographic and clinical characteristics of the GCP are summarised in Table 2.

**Table 2:** Demographic and clinical characteristics of the GCP.

Variable	Category	Frequency (n)	Percentage
Age group of the child (in years)	2–6	3	12%
	7–11	7	28%
	12–14	7	28%
	15–18	8	32%
Level of CP	Level 1	2	8%
	Level 2	9	36%
	Level 3	10	40%
	Level 4	3	12%
	Level 5	1	4%
IQ Level	50–60	1	4%
	61–70	7	28%
	71–80	10	40%
	81–90	7	28%
Duration of Treatment (years)	0–5	2	8%
	6–10	8	32%
	11–15	11	44%
	>15	4	16%
Response to CP Management	Improvement	24	96%
	No improvement	1	4%
Other Medical Conditions	NIL	25	100%

### 3.3 Participants’ engagement with the intervention

All participants completed the three-month follow-up. The majority reported referring to the IEC booklet multiple times, and no challenges were reported in understanding the material. No dropouts or adverse experiences were reported during the intervention.

### 3.4 Effect of the RHE intervention on caregivers’ knowledge related to the reproductive health of GCP

The caregivers' knowledge of GCP's reproductive health before and after the intervention are depicted in Fig. 1. A significant improvement was observed following the intervention with mean knowledge score increased from 41.2 ± 4.66 pre-intervention to 62.3 ± 1.77 post-intervention (p < 0.001). The magnitude of change corresponded to a very large effect size (Cohen's d = 5.43).

**Fig. 1:**
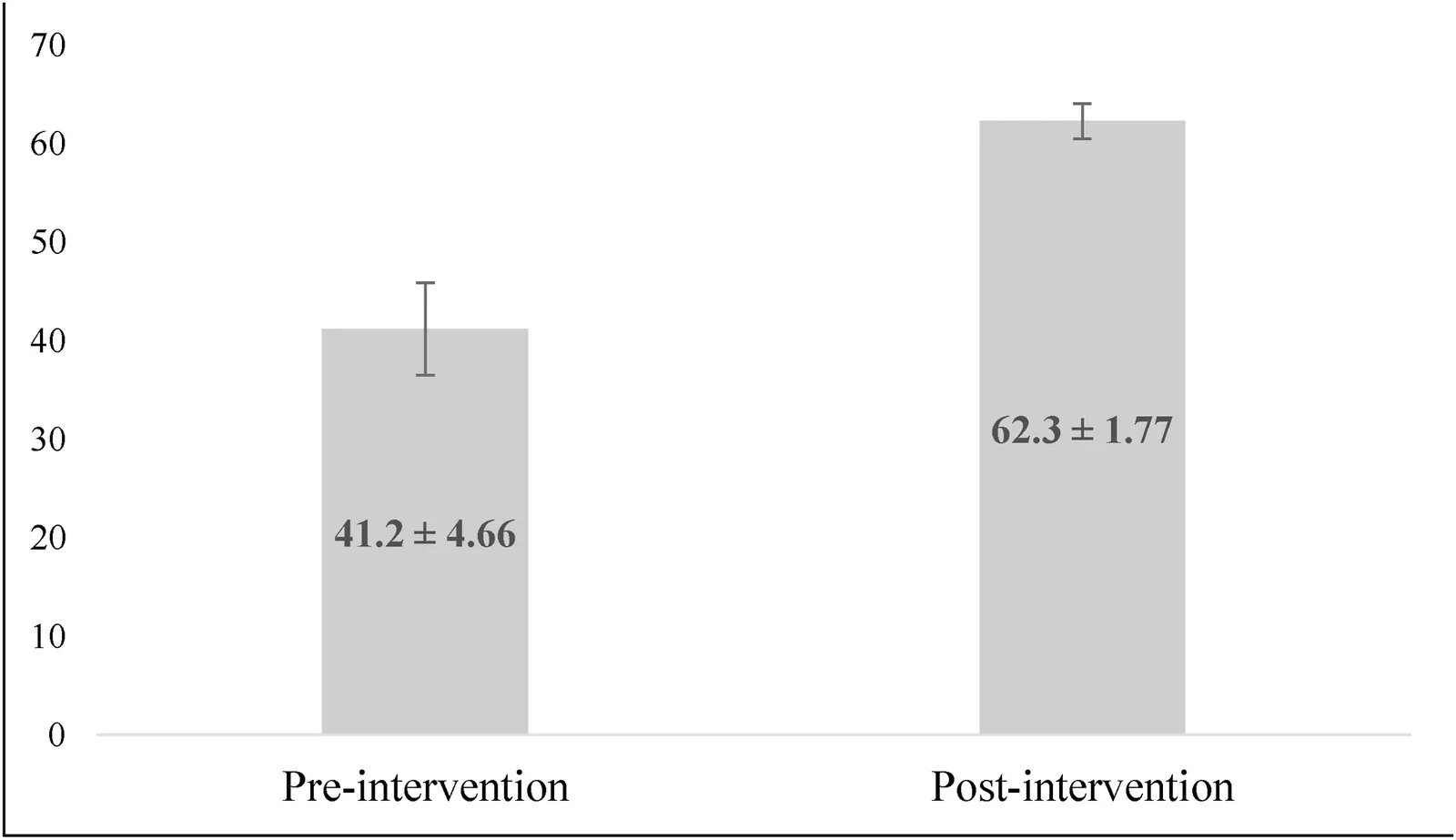
Mean knowledge score pre-and post-intervention (mean ± SD) Knowledge score: 0-65.

A detailed item-level comparison of pre- and post-intervention knowledge, along with paired *t*-test results and effect sizes, is presented in Table 3.

**Table 3:** Paired *t*-test analysis comparing the pre-post-intervention scores of caregivers’ knowledge in reproductive health of GCP.

Item No.	Pre-test Mean ± SD	Post-test Mean ± SD	Mean Difference	SE Difference	95% CI (Lower, Upper)	t-value	df	p-value
1. Menstruation is beneficial for the overall health of girls, including those with developmental delays.	4.12 ± 0.83	5.00 ± 0.00	0.88	0.167	[1.224, 0.536]	5.28	24	< 0.001
2. Regular menstruation helps in maintaining strong bones and a healthy heart.	2.04 ± 1.02	4.76 ± 0.44	2.72	0.196	[3.124, 2.316]	13.88	24	< 0.001
3. Menstruation indicates good functioning of uterus and ovaries.	3.16 ± 0.99	4.72 ± 0.46	1.56	0.201	[1.974, 1.146]	7.77	24	< 0.001
4. Suppressing or stopping menstruation can negatively impact a girl's physical and mental health.	2.56 ± 0.82	4.84 ± 0.37	2.28	0.147	[2.584, 1.976]	15.47	24	< 0.001
9. It is common and normal for girls to experience minimal stomach pain and body ache during menstruation.	3.64 ± 0.95	4.76 ± 0.44	1.12	0.145	[1.420, 0.820]	7.72	24	< 0.001
10. Consulting a doctor is advisable if a girl experiences severe menstrual pain.	2.68 ± 0.85	4.76 ± 0.44	2.08	0.140	[2.370, 1.790]	14.81	24	< 0.001
11. Providing information about menstrual pain can help relieve parents' concerns.	2.88 ± 0.78	4.68 ± 0.48	1.80	0.153	[2.115, 1.485]	11.78	24	< 0.001
12. Parents of children with developmental delays should attend social functions without worrying about public judgment.	2.56 ± 1.04	4.64 ± 0.49	2.08	0.152	[2.393, 1.767]	13.70	24	< 0.001
13. It is important to educate children with developmental delays about personal safety and recognizing inappropriate behaviour.	3.76 ± 0.72	5.00 ± 0.00	1.24	0.145	[1.539, 0.941]	8.57	24	< 0.001
17. (REVERSE CODED) Irregular menstrual cycles in girls with cerebral palsy are directly related to their condition.	4.32 ± 0.75	4.92 ± 0.28	0.60	0.115	[0.838, 0.362]	5.20	24	< 0.001
19. Irregular periods can be effectively managed with medication.	2.56 ± 0.87	4.80 ± 0.41	2.24	0.145	[2.539, 1.941]	15.48	24	< 0.001
20. Cerebral palsy is not hereditary.	4.04 ± 0.68	4.92 ± 0.28	0.88	0.120	[1.128, 0.632]	7.33	24	< 0.001
21. Cerebral palsy does not affect the ability to have normal and healthy babies.	2.84 ± 0.80	4.48 ± 0.71	1.64	0.151	[1.953, 1.327]	10.83	24	< 0.001

### 3.5 Effect of the RHE intervention on reproductive health-related caregiving intention among caregivers of GCP

The caregivers' reproductive health-related caregiving intentions before and after the intervention are depicted in Fig. 2. Significant improvements were observed in reproductive health-related caregiving intention among the participants, with the mean caregiving intention score increased from 41.2 ± 4.02 to 57.6 ± 1.78 (p < 0.001), with a very large effect size (Cohen's d = 5.22). A detailed item-level comparisons of pre- and post-intervention caregiving intention, along with paired *t*-test results and effect sizes, are presented in Table 4.

**Fig. 2:**
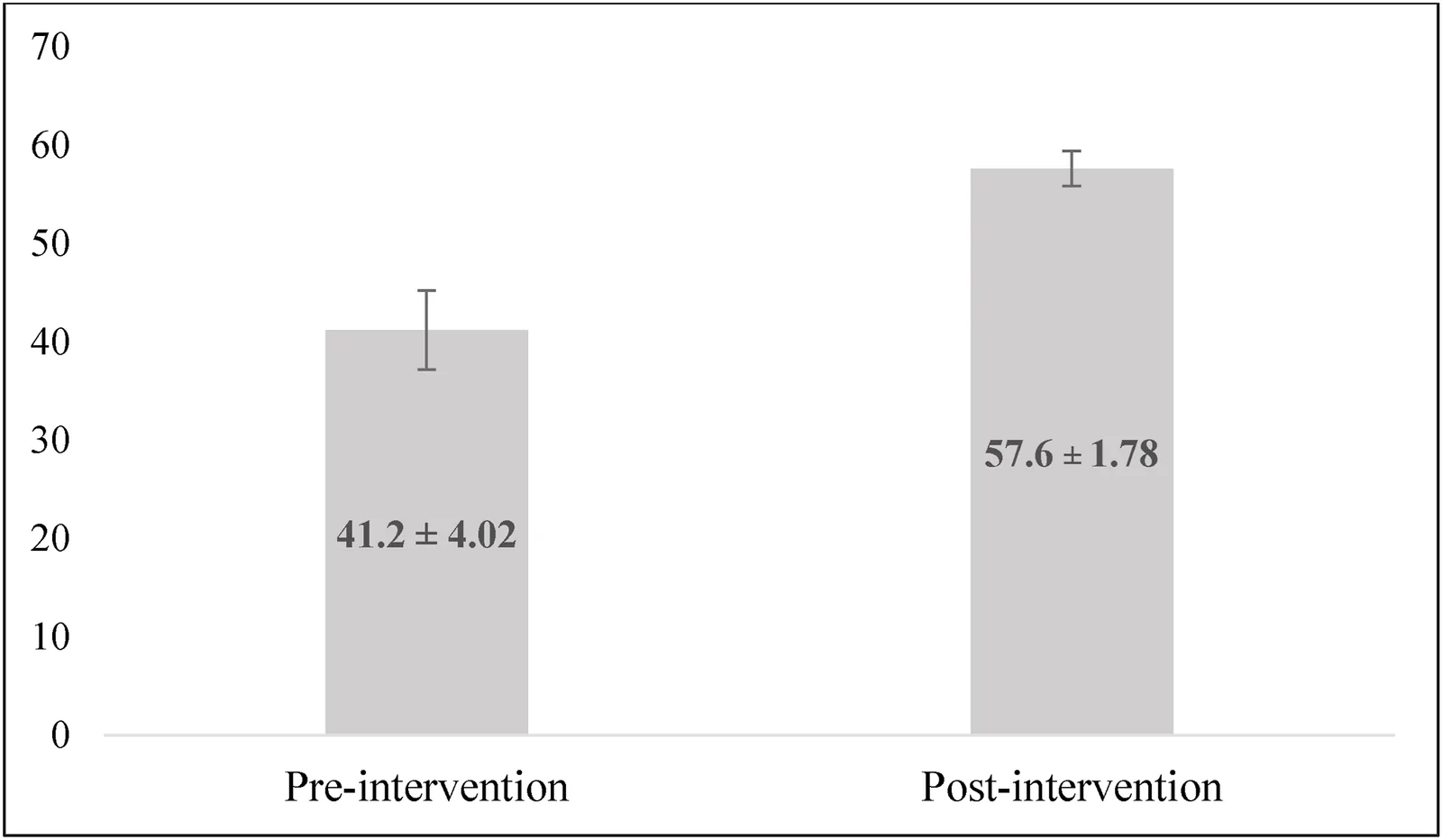
Mean caregiving intention score pre-and post-intervention (mean ± SD) Caregiving intention score: 0-60.

**Table 4:** Paired *t*-test analysis comparing pre- and post-intervention scores of reproductive health-related caregiving intention among caregivers of GCP.

Item No.	Pre-test Mean ± SD	Post-test Mean ± SD	Mean Difference	SE Difference	95% CI (Lower, Upper)	t-value	df	p-value
5. I will help my daughter with developmental delays learn to use sanitary pads independently for better menstrual hygiene.	3.84 ± 0.80	4.84 ± 0.37	1.000	0.1414	(1.291, 0.709)	7.07	24	< 0.001
6. I will ensure that my daughter uses sanitary pads during menstruation to maintain proper hygiene.	3.60 ± 0.71	4.84 ± 0.37	1.240	0.1046	(1.457, 1.023)	11.86	24	< 0.001
7. I will avoid the use of cotton cloth for my daughter during menstruation to reduce the risk of infection.	3.60 ± 0.65	4.92 ± 0.28	1.320	0.1254	(1.579, 1.061)	10.52	24	< 0.001
8. I will provide longer and more absorbent pads to my daughter when she experiences heavy menstrual bleeding.	4.00 ± 0.71	4.88 ± 0.33	0.880	0.1200	(1.128, 0.632)	7.33	24	< 0.001
14. I will teach my daughter to raise an alarm if she experiences any form of inappropriate behavior.	4.08 ± 0.76	5.00 ± 0.00	0.920	0.1519	(1.235, 0.605)	6.06	24	< 0.001
15. I will seek medical consultation if my daughter experiences heavy menstrual bleeding.	3.72 ± 0.54	4.84 ± 0.37	1.120	0.0663	(1.258, 0.982)	16.88	24	< 0.001
16. I will seek medical consultation if my daughter experiences irregular menstrual bleeding.	2.44 ± 0.77	4.88 ± 0.33	2.440	0.1641	(2.779, 2.101)	14.87	24	< 0.001
18. I will not hesitate to use hormonal tablets for my daughters menstrual issues.	2.60 ± 0.58	4.52 ± 0.51	1.920	0.1281	(2.185, 1.655)	14.99	24	< 0.001
22 I intend to support my daughter in choosing a supportive life partner if she decides to get married in the future.	2.48 ± 1.08	4.64 ± 0.49	2.160	0.1796	(2.530, 1.790)	12.02	24	< 0.001
23 I will ensure that my daughter receives proper medical care during pregnancy, if she chooses to become a mother.	3.64 ± 0.81	4.68 ± 0.56	1.040	0.0909	(1.226, 0.854)	11.44	24	< 0.001
24. After consulting the doctor, I have started considering the possibility of my daughter getting married and having children.	4.00 ± 0.87	4.92 ± 0.40	0.920	0.1281	(1.186, 0.654)	7.18	24	< 0.001
25. I actively plan to support my daughter in leading a fulfilling life, including the possibility of marriage and motherhood.	3.20 ± 0.82	4.68 ± 0.48	1.480	0.1306	(1.749, 1.211)	11.33	24	< 0.001

The results of paired t-tests assessing changes in mean knowledge and caregiving intention scores pre- and post-intervention are depicted in Table 5.

**Table 5:** Paired t-tests assessing changes in mean knowledge and caregiving intention scores pre- and post-intervention Knowledge score: 0-65 Caregiving intention score: 0-60.

Outcome	Pre-test Mean ± SD	Post-test Mean ± SD	Mean Difference	95% CI of Mean Difference	*t* (df)	*p*-value	Effect size^a^
**Knowledge**	41.2 ± 4.66	62.3 ± 1.77	−21.1	−22.7 to −19.5	−27.2 (24)	<0.001	−5.43
**Caregiving intention**	41.2 ± 4.02	57.6 ± 1.78	−16.4	−17.7 to −15.1	−26.1 (24)	<0.001	−5.22

a Cohen's d.

## 4 Discussion

The findings of this study provide preliminary evidence on a reproductive health education intervention, integrated into routine CP care in resource-constrained settings. The intervention required minimal time (20-25 min), no additional infrastructure and was delivered during routine CP consultation. Such interventions could be delivered by a range of trained personnel, including healthcare providers, nurses, and community health workers, supporting task-sharing approaches.

The relatively lower levels of knowledge and caregiving intention observed in the pre-intervention survey indicate that menstrual and reproductive health remain poorly addressed aspects of care for GCP. Though caregivers have frequent interactions with healthcare providers, reproductive health-related topics may not always be discussed due to time constraints in the clinical setting or due to the prevailing “taboo” related to the sexual and reproductive health of individuals with intellectual disabilities. [22] Stigma surrounding menstruation and the sexuality of disabled girls may further discourage open discussions. [23] Consequently, caregivers remain uncertain about managing menstruation, maintaining menstrual hygiene and addressing other reproductive health concerns of their daughters with disabilities.

Similar knowledge gaps among caregivers of girls with disabilities have been identified in different settings. For instance, research conducted in Maharashtra, India, identified a poor understanding of proper menstruation management among caregivers of girls with developmental disabilities. [24] Similarly, in Turkey, a low level of knowledge regarding menstrual hygiene management among caregivers was observed. [25] Additionally, it was identified that caregivers rely on informal social networks to learn about the reproductive health of their daughters with disabilities. [26] While these networks can provide emotional and practical support, they may also contribute to the circulation of incomplete or inaccurate information. This underscores the need to provide caregivers with structured, culturally appropriate, right education. The need for better access to sexual and reproductive health-related information among caregivers of girls with intellectual disabilities has been well documented. [27] As reported by Magai et al., there remains a paucity of structured guidance addressing reproductive health education for the paediatric population with disabilities in the LMIC context, underscoring the need for targeted intervention. [28]

Item-level analysis indicated improved understanding of several key concepts following the intervention. Caregivers demonstrated improved understanding of the importance of educating girls with developmental disabilities about personal safety and identifying inappropriate behaviour. In addition, the intervention helped correct common misconceptions, such as the belief that cerebral palsy is hereditary or that it affects a girl's ability to have healthy children (Table 3). Post-intervention responses indicated stronger commitment among caregivers to help their daughters use sanitary pads independently, ensure consistent pad use, and avoid using cloths to reduce the risk of infection. They also showed readiness to consult a doctor during irregular bleeding and for the use of hormonal methods if required. Additionally, caregivers reported stronger commitment to teaching personal safety skills and expressed more supportive attitudes toward their daughters' prospects, including marriage, pregnancy, and motherhood (Table 4). These findings indicate measured changes in knowledge and reproductive-health-related caregiving intentions post-intervention. However, it should be interpreted with caution as there were no comparison group. Additionally, the intervention demonstrated very large effect size (Cohen's d > 5). A large effect size may be explained by substantial differences between pre- and post-knowledge and caregiving intention scores, as well as by the study's limited sample size (n = 25). Furthermore, because the outcomes were based on self-reported measures, social desirability bias could not be entirely ruled out and may have contributed to the observed differences between pre-test and post-test scores.

The observed improvement in knowledge and caregiving intention is consistent with previous studies demonstrating the effectiveness of interventions targeting caregivers of children with disabilities. For example, a supportive intervention for mothers of girls with cerebral palsy significantly improved knowledge and caregiving intentions related to menstruation management in Egypt. [20] Similarly, a group training in Iran for caregivers was found effective in enhancing knowledge, attitudes, and confidence in providing sexual and reproductive care for intellectually disabled girls. [18] Additionally, in Turkey, a simulation-based education targeting both girls with intellectual disabilities and their mothers significantly enhanced the knowledge and skills of menstruation management and reduced anxiety in both groups. [19] These interventions typically emphasise interactive learning, discussions and culturally appropriate communication, which may enhances caregivers’ understanding of sensitive topics. In the present study, the use of educational materials in the local language and personalised sessions may have facilitated greater engagement and improved outcomes. However, it is important to acknowledge that improvements in knowledge and caregiving intention do not automatically translate into behaviour changes. Health behaviour theories, such as the theory of planned behaviour, suggest that behavioural outcomes are influenced by factors including attitude, subjective norms, and perceived behaviour control. [29] Therefore, though the observed increase in knowledge and caregiving intentions is encouraging, further research should confirm whether it resulted in long-term improvement in behaviour change and better caregiving practices among caregivers of GCP.

The findings of this study provide preliminary evidence for clinical integration. Integrating reproductive health education into GCP's routine clinical services could help address the information needs of caregivers who may otherwise remain uninformed, underinformed, or misinformed. Training healthcare professionals to deliver disability-sensitive reproductive health education to caregivers may strengthen the health system's capacity to support caregivers and GCP. In addition, community-based adaptation of this model through special school staff or Anganwadi workers (grassroots-level community health workers in India) can further enhance the scalability and accessibility of such interventions to a wider community of caregivers.

### 4.1 Strengths, limitations and implications for future research

The present study contributes to the limited evidence base on effective interventions to enhance caregivers' menstrual and reproductive health knowledge among GCP in low- and middle-income countries. The utilisation of tailored, culturally sensitive RHE, adapted to the participants' local languages, and the one-on-one session allowed participants to clarify doubts and discuss sensitive concerns, increasing engagement and trust. The achievement of complete retention at the three-month follow-up and the demonstrated large, statistically significant improvements in both knowledge and caregiving intention support the intervention's positive effect among caregivers of GCP.

Nevertheless, certain limitations should be acknowledged while interpreting the findings.

First, a single-arm pretest-post-test design without a concurrent control group limits the ability to establish a causal relationship between the intervention and the observed improvement.

Second, since this study was conducted as part of a research project for a master's degree thesis, which was restricted to a period of six months and the study was conducted in the cerebral palsy clinic, which operates only once a week, it was not feasible to recruit participants based on a predetermined sample size using a priori power calculation. Hence, the study was conducted among only 25 participants, who were available during the study period. This small sample size restricts the generalizability of the findings, may have contributed to the unusually large observed effect size and may limit the validity of the study findings.

Third, although reliability was calculated for the pre-test questionnaire, it could not be calculated for the post-test questionnaire because several items showed no variance (ceiling effect). In addition, the tool's reliability at pre-test was below the commonly recommended threshold of 0.70, indicating limited internal consistency. Therefore, the findings should be interpreted with caution, as measurement error may have influenced the observed changes in scores.

Fourth, since the study was conducted within a limited six-month timeframe and involved a relatively difficult-to-access population in a setting with limited operating hours, a separate pilot study was not feasible, as it would require a different set of participants.

Fifth, although measures were taken to minimise the researcher-related bias and social desirability biases, having the same researcher deliver the intervention and collect outcome data may increase the risk of measurement and social desirability biases. Additionally, since the outcome measures were self-reported, social desirability bias could not be entirely ruled out and may have contributed to the observed differences between pre-test and post-test scores.

Sixth, although the intervention was developed based on the findings (suggestions and recommendations given by the caregivers) of a mixed-methods study conducted by the authors, it was not explicitly grounded in a specific theoretical or conceptual framework. This may have limited the systematic development and theoretical interpretation of the intervention.

Future studies should employ a randomised controlled design with a concurrent control group to strengthen the causal inference and to distinguish intervention-related changes from the changes attributed to external factors. Studies should also include a large sample with priori calculated sample size to generalize the findings and to provide more precise estimates of the intervention effectiveness. Given the small sample and the unusually large effect size observed in the present study, the evidence must be interpreted with caution and must be considered as preliminary evidence. Future research should conduct a formal psychometric evaluation of the instrument, including reliability and formal piloting, before undertaking a larger controlled study. To minimise potential researcher-related and social desirability biases, future studies should consider having intervention delivery and outcome assessment performed by different researchers. The observed improvements in intention should not be interpreted as evidence of actual behavioural change, and future studies should incorporate objective or behavioural measures and longer follow-up to determine whether improvements in caregiving intention translate into sustained changes in caregiving practices. Overall, this study must be considered a pilot study, and the evidence is preliminary. Future controlled studies with a large sample, longer follow-up, and improved measurement procedures are required to generalize the findings.

## Ethics statement

The study was conducted in accordance with the Indian Council of Medical Research (ICMR) National Ethical Guidelines for Biomedical and Health Research Involving Human Participants (2017). The study was approved by the Institutional Ethics Committee [418/2024]. The study was also registered under the Clinical Trial Registry of India (CTRI) – Registration No. CTRI/2024/12/078042.

## Data availability statement

The datasets generated and/or analysed during the current study are not publicly available but are available from the corresponding author on reasonable request.

## Author contributions

CB: Conceptualisation, data curation, formal analysis, investigation, methodology, project administration, software, writing-original draft, writing-reviewing and editing. AP: methodology, writing-original draft, writing-reviewing and editing, validation HS: Methodology, investigation, supervision, validation, writing-reviewing and editing. APR: Conceptualisation, formal analysis, methodology, supervision, validation, visualisation, project administration, writing, reviewing and editing.

## Funding

The authors received no funding for this study.

## Declaration of competing interest

The authors declare that they have no known competing financial interests or personal relationships that could have appeared to influence the work reported in this paper.

## References

[1] Children with disabilities - UNICEF DATA. https://data.unicef.org/topic/child-disability/overview/.

[2] Patel D.R., Neelakantan M., Pandher K., Merrick J. (2020). Cerebral palsy in children: a clinical overview. Transl. Pediatr..

[3] McIntyre S., Goldsmith S., Webb A. (2022). Global prevalence of cerebral palsy: a systematic analysis. Dev. Med. Child Neurol..

[4] Chauhan A., Singh M., Jaiswal N., Agarwal A., Sahu J.K., Singh M. (2019). Prevalence of cerebral palsy in Indian children: a systematic review and meta-analysis. Indian J. Pediatr..

[5] Cerebral palsy: what it Is, causes, symptoms & treatment. https://my.clevelandclinic.org/health/diseases/8717-cerebral-palsy.

[6] Cerebral palsy – symptoms - NHS. https://www.nhs.uk/conditions/cerebral-palsy/symptoms/.

[7] Rausch R., Chahin S., Miller C. (2025). The mental health of children with cerebral palsy: a review of the last five years of research. J. Clin. Med..

[8] Elkholi S.M., Awad S.S., Alotaibi M.H. (2024). Neglect of psychological care for children with cerebral palsy and their families and its impact on their occupational engagement in Saudi Arabia. Medicina.

[9] Cerebral palsy | national institute of neurological disorders and stroke. https://www.ninds.nih.gov/health-information/disorders/cerebral-palsy.

[10] Power R., Wiley K., Muhit M. (2020). “Flower of the body”: menstrual experiences and needs of young adolescent women with cerebral palsy in Bangladesh, and their mothers providing menstrual support. BMC Womens Health.

[11] Rao A.P., Shah H., Guruvare S., Guddattu V. (2019). Growth, sexual development and menstrual issues among girls with cerebral palsy – a cross sectional study in a tertiary care centre. Clin Epidemiol Glob Health.

[12] Sarmiento C.A., Glaros C., Sanders J.S., Wyrwa J.M., Holliman B.D., Brenner L.A. (2024). “We do it all”: a qualitative exploration of the caregiver role for young adults with cerebral palsy. Health Care Transitions.

[13] Liu F., Shen Q., Huang M., Zhou H. (2023). Factors associated with caregiver burden among family caregivers of children with cerebral palsy: a systematic review. BMJ Open.

[14] Rao A.P., Shah H., Guruvare S., Gore M., Nayar K.R., Vijayan B. (2021). Perspectives of caregivers on pubertal changes among young adolescent girls with cerebral palsy: a qualitative analysis. Disability, CBR & Inclusive Development.

[15] Menon P., Sivakami M. (2019). Exploring parental perceptions and concerns about sexuality and reproductive health of their child with intellectual and developmental disability (IDD) in Mumbai. Front. Sociol..

[16] Fatima S., Saeed M., Pandey N., Sarraf S.R. (2024). Dilemmas being encountered by primary caregivers in handling the hygiene of menstruating girls with intellectual disability (ID): an exploratory study. Educational Administration: Theor. Pract..

[17] Bhosale M.M., Prabhakaran A., Rao A.P. (2025). A qualitative study on the experiences of caregivers on menstrual management of adolescent girls with developmental disabilities in selected special schools of karveer taluka, Maharashtra. BMC Womens Health.

[18] Goli S., Noroozi M., Salehi M. (2021 18:1. 2021). Comparing the effect of two educational interventions on mothers' awareness, attitude, and self-efficacy regarding sexual health care of educable intellectually disabled adolescent girls: a cluster randomized control trial. Reprod. Health.

[19] Kaçan H., Birol Z. (2024). The impact of an applied menstrual self-care education program given to adolescents with intellectual disabilities and their mothers on their knowledge, skills, and anxiety levels. Int. J. Dev. Disabil..

[20] Draz S.F., ATA Elsharkawy (2021). Effect of supportive educational intervention for mothers of female adolescents with cerebral palsy on their caring practices. Evidence-Based Nursing Research.

[21] Gross motor function classification system (GMFCS) | cerebral palsy alliance research foundation. https://cparf.org/what-is-cerebral-palsy/severity-of-cerebral-palsy/gross-motor-function-classification-system-gmfcs/.

[22] Pérez-Curiel P., Vicente E., Morán M.L., Gómez L.E. (2023). The right to sexuality, reproductive health, and found a family for people with intellectual disability: a systematic review. Int. J. Environ. Res. Publ. Health.

[23] Wilbur J., Kayastha S., Mahon T. (2021). Qualitative study exploring the barriers to menstrual hygiene management faced by adolescents and young people with a disability, and their carers in the Kavrepalanchok district, Nepal. BMC Public Health.

[24] Bhosale M.M., Prabhakaran A., Rao A.P. (2025). A qualitative study on the experiences of caregivers on menstrual management of adolescent girls with developmental disabilities in selected special schools of Karveer Taluka, Maharashtra. BMC Womens Health.

[25] Elma C., Bilge Ç. (2025). Forgotten hygiene: exploring menstrual self-care skills and parents awareness in adolescents with intellectual disability. Int. J. Dev. Disabil..

[26] Gray S.H., Wylie M., Christensen S., Khan A., Williams D., Glader L. (2021). Puberty and menarche in young females with cerebral palsy and intellectual disability: a qualitative study of caregivers' experiences. Dev. Med. Child Neurol..

[27] Powell R.M., Parish S.L., Mitra M., Rosenthal E. (2020). Role of family caregivers regarding sexual and reproductive health for women and girls with intellectual disability: a scoping review. J. Intellect. Disabil. Res..

[28] Magai D.N., Berman B.D., Mutua A.M. (2025). Scoping review of evidence-based practice guidelines for the evaluation and care of young children with developmental disabilities in LMIC settings: evidence for action. BMJ Paediatr Open.

[29] Ajzen I. (1991). The theory of planned behavior. Organ. Behav. Hum. Decis. Process..

